# Age at Diagnosis of Cataract and Subsequent Risk of Dementia

**DOI:** 10.1002/alz.083581

**Published:** 2025-01-03

**Authors:** Chaofan Geng

**Affiliations:** ^1^ Xuanwu hospital, beijing, beijing China

## Abstract

**Background:**

Epidemiological evidence regarding the association between cataract onset age and risk of incident dementia remains unexplored. To examine whether age at cataract diagnosis is associated with risk of incident dementia and its subtypes.

**Methods:**

This prospective, population‐based cohort study utilized data from the UK Biobank that collected baseline information between 2006 and 2010. A total of 442,376 participants who were diagnosed with dementia at baseline, had missing covariate data, or had dementia before the onset of cataracts during a median follow‐up of 12.6 years were excluded from the analysis. Cox regression models and propensity score matching was ultimately used to analyze the associations between cataracts and their age of onset with subsequent all‐cause dementia, Alzheimer’s disease (AD), and vascular dementia (VD).

**Results:**

Upon comparison with subjects unaffected by cataract, patients afflicted with cataract exhibited a significantly elevated risk of all‐cause dementia ((hazard ratio [HR]: 1.78, 95% confidence interval [CI]: 1.62‐1.98), AD (HR:1.79, 95% CI: 1.92‐2.48) and VD (HR: 1.78, 95% CI: 1.54‐2.04). After propensity score matching, compared with individuals without cataract, individuals with cataract diagnosed at younger than 60 years had the highest HR for developing VD (adjusted HR = 3.73, 95%CI: 1.25‐11.12, p‐value = 0.018).

**Conclusion:**

In this prospective cohort study, an earlier onset of cataracts was found to be associated with an increased risk of subsequent VD, highlighting the importance of monitoring cataract patients, particularly those diagnosed at age less than 60 years, for the occurrence of VD, providing new ideas for the clinical prevention and intervention of VD.

